# Challenge levels of everyday technologies as perceived over five years by older adults with mild cognitive impairment

**DOI:** 10.1017/S1041610218000285

**Published:** 2018-04-04

**Authors:** Annicka Hedman, Anders Kottorp, Ove Almkvist, Louise Nygård

**Affiliations:** 1 Department of Neurobiology, Care Sciences and Society, Division of Occupational Therapy, Karolinska Institutet, Huddinge, Sweden; 2 Faculty of Health and Society, Malmö University, Malmö, Sweden; 3 Department of Neurobiology, Care Sciences and Society, Division of Clinical Geriatrics, Karolinska Institutet, Huddinge, Sweden; 4 Department of Psychology, Stockholm University, Stockholm, Sweden

**Keywords:** longitudinal studies, dementia, activities of daily living (ADLs)

## Abstract

**Background:**

In clinical practice, efficient and valid functional markers are needed to detect subtle cognitive and functional decline in mild cognitive impairment (MCI). This prospective study explored whether changes in perceived challenge of certain everyday technologies (ETs) can be used to detect signs of functional change in MCI.

**Methods:**

Baseline and five-year data from 37 older adults (mean age 67.5 years) with MCI regarding their perceived ability to use ET were used to generate Rasch-based ET item measures reflecting the relative challenge of 46 ETs. Actual differential item functioning in relation to time was analyzed based on these item measures. Data collection took place in 2008–2014.

**Results:**

Seven (15%) of the ETs included were perceived to be significantly more challenging to use at year five compared to at baseline, while 39 ETs (85%) were perceived to be equally challenging to use, despite the fact that the participants’ perceived ability to use ET had decreased. Common characteristics among the ETs that became more challenging to use could not be identified. The dropout rate was 43%, which limits the power of the study.

**Conclusions:**

Changes in the perceived challenge of ETs seem to capture functional change in persons with cognitive decline. Both easier and more challenging ETs typically used at home and in society need to be addressed to capture this functional change because significant changes occurred among ETs of all challenge levels and within all types of ETs.

## Introduction

Mild cognitive impairment (MCI) is a heterogeneous clinical syndrome that lies between the cognitive functioning in normal aging and the early stage of dementia (Petersen *et al*., 2014). Core clinical criteria in former and current major definitions of MCI include roughly preserved independence in functional abilities despite some objectively verified cognitive impairment and self- or informant-reported cognitive symptoms, thus not dementia (Petersen, 2004; Winblad *et al*., 2004; American Psychiatric Association, 2013). This description overlaps with criteria intended to describe preclinical Alzheimer's disease (AD) (Albert *et al*., 2011). To date, no effective treatment of MCI is available (Kane *et al*., 2017). Nevertheless, early detection of morbid cognitive and functional decline remains important; in the current situation in order to enable adequate and targeted support to handle the consequences of such declines in everyday life, and in the future to identify the optimal therapeutic window (Belleville *et al*., 2014). In clinical practice, efficient and valid functional markers are needed that capture the subtle cognitive and functional decline in MCI. Impaired ability to manage finances and medications (Arrighi *et al*., 2013) and withdrawal from participating in outings and other leisure activities (Arrighi *et al*., 2013; Hedman *et al*., 2016) have been suggested as early functional signs of cognitive change. In addition, changes in the ability to use everyday technologies (ETs), i.e. the wide range of technical objects and services that are commonly present and used in our everyday lives at home and in the community, have proven to be one such potential marker (Malinowsky *et al*., 2010; Nygård *et al*., 2012; Malinowsky *et al*., 2017). ET use can, for example, be studied by assessing the ability of individuals to use specific ETs or by focusing on the challenge level of specific ETs as perceived by users. This prospective five-year study explored whether the challenge level of certain ETs is more sensitive and well suited to detecting signs of functional change in MCI.

Cognitive ability has been shown to affect how many types of ETs people use (Czaja *et al*., 2006) and how efficient this use is (Slegers *et al*., 2009). On a group level, both perceived (Nygård *et al*., 2012) and observed (Malinowsky *et al*., 2010) ability to use ETs are significantly lower in persons with MCI compared to in persons with no known cognitive impairment, and even lower in persons with dementia. Ability in ET use as well as amount of ETs used have shown a potential to differentiate groups with different needs of assistance among persons with cognitive impairment (Ryd *et al*., 2016). Furthermore, a declining pattern regarding the ability to use ETs and activity involvement during the years following the detection of MCI might be predictive of future dementia (Hedman *et al*., 2017a).

The cognitive decline in MCI includes impaired memory related to recollective/associative abilities and short-term retention, impaired executive functions in new or demanding tasks, and reduced attentional control (Belleville *et al*., 2014), which together with visuo-spatial function all exemplify cognitive abilities needed when using ETs such as coffee makers, cell phones, and cash machines (ATMs). Performance skills, i.e. observable actions when performing everyday activities, often involved when managing ETs and known to be, especially challenging are choosing the correct button or command, identifying the services or functions of the ET, and performing actions in a logical sequence (Malinowsky *et al*., 2011). We also know that certain ET characteristics make their use more challenging for persons with and without cognitive impairments. ETs demanding a high frequency of performance skill actions, requiring use of more difficult performance skills, failing to provide feedback related to a variety of sensory functions (e.g. visual, auditory, and tactile), having complex design, and typically being infrequently used, pose higher levels of challenge on the users (Patomella *et al*., 2011, 2013). Thus, complexity in process might be the common denominator challenging both cognition and ET use. Cross-sectional research has suggested that domestic ETs such as coffee makers, stoves, and microwave ovens are generally perceived and observed to be easier to use, while information and communication technologies (ICT) like cell phones and computers are more challenging (Patomella *et al*., 2011; Malinowsky *et al*., 2013). However, longitudinal studies examining how the perceived challenge of specific ETs develops in persons with cognitive decline are lacking. Identifying ETs whose challenge level has the potential to detect functional change might be highly interesting for ensuring targeted support at an early stage. Therefore, we examined how older adults perceived the challenge levels of commonly used ETs at the time of MCI detection and five years later, with the aim to investigate whether the use of certain ETs is more sensitive to cognitive decline.

## Methods

### Sample

**Figure 1.**
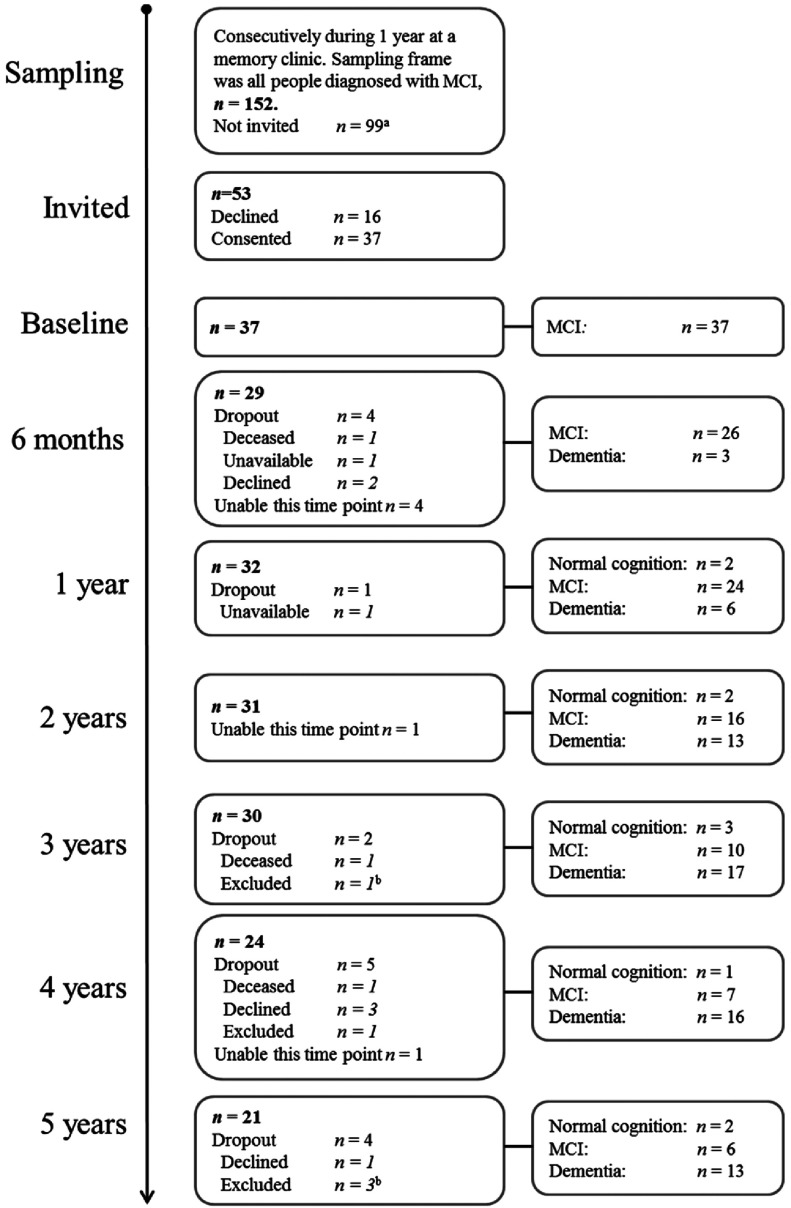
Overview of sampling, data available and missing, and diagnostic information at the seven follow-up occasions.

Participants were older adults with MCI diagnosed and recruited at a specialized outpatient memory clinic in Stockholm between April 2008 and May 2009 (Table 1). The inclusion criteria were: (a) fulfilled criteria for MCI as proposed by (Petersen, 2004), i.e. self-rated and clinically verified cognitive decline, no dementia, and essentially intact basic and instrumental activities of daily living; (b) at least 55 years old; (c) being a user of ETs; (d) the ability to take part in data collection in Swedish; (e) no cognitive comorbidities; and (f) no severe problems with hearing or vision that could not be compensated for. The Regional Ethics Committee in Stockholm approved the study, and informed consent was obtained from all participants. Figure 1 provides an overview of the sampling and of the data that were available and missing at the seven follow-up occasions over the five-year period. As the inclusion ended 2009, the final 5-year-follow-ups took place in 2014.

**Table 1.** Participant characteristics at inclusion (*n* = 37)

variable	value
Sex, *n* (%)	
Female	18 (49)
Male	19 (51)
Age, years	
Mean (SD)	67.4 (7.5)
Min-max	56–82
Living condition, *n* (%)	
Cohabiting	28 (76)
Living alone	9 (24)
Education, years	
Mean (SD)	12.6 (3.3)
Min-max	6–20
ETUQ person measure	
Mean (SD)	54.6 (2.8)
Min-max	48.5–60.6
MMSE	
Median (IQR)	28 (2)
Min-max	19–30

*Note*: Mean and SD are presented for normally distributed data, while median and IQR are given for skewed data. ETUQ: Everyday Technology Use Questionnaire, where higher person measure indicates higher perceived ability in ET use (Nygård *et al*., 2012). MMSE: Mini-Mental State Examination (0–30), where higher score indicates better cognitive status (Folstein *et al*., 1975).

*Notes:*^a^Those who did not meet inclusion criteria (*n* = 71) were not invited. Other reasons (*n* = 28) for not being invited included, for example, social problems and living too far away from the clinic for practical data collection.

^b^Exclusion based on ethical considerations.

### Data collection

In the present study, we used baseline and five-year data because a previous study using the same sample (Hedman *et al*., 2017b) had shown a significant decrease in perceived ability to use ETs only when comparing year five and baseline, and not between consecutive time points. The first author and five research assistants conducted the structured face-to-face interviews, most often in the homes of the participants. Those who so wished were accompanied by a significant other for support, but we based the scoring on the participants’ answers. To capture longitudinal changes in the perceived level of challenge of a range of ETs of varying difficulty, the Everyday Technology Use Questionnaire (ETUQ) was used (Rosenberg *et al*., 2009). The ETUQ includes 92 ETs commonly used by older adults inside and outside the home. Each ET perceived as relevant by the person is scored on a 6-step scale, rating the person's perceived ability to use it (Table 2). In the ETUQ, a relevant ET is defined as an ET that the person has access to and has used in the past, currently is using, or intends to start using. In this study, both *person measures* and *item measures* were used (further explained in the data analysis section). The ETUQ has shown acceptable psychometric properties (unidimensionality, rating scale validity, and person response validity) in studies including older adults with cognitive impairments (Rosenberg *et al*., 2009; Nygård *et al*., 2012). Additionally, cognitive function was assessed using the Mini-Mental State Examination (MMSE) (Folstein *et al*., 1975), and we obtained diagnostic information from medical files at the memory clinic.

**Table 2.** Description of the scale steps in the ETUQ

score	description
6	The ET is used with no hesitation or difficulty at all
5	The ET is used with minor hesitation or difficulty
4	The ET is used with frequent/major difficulties
3	The ET is sometimes/partly used together with another person
2	The ET is only used together with another person
1	The ET is not used anymore, or has not come into use, even if it is available and relevant for the person
No score	Non-relevant

### Data analysis

We used a Rasch model to transform the ordinal ETUQ scores into relative linear measures expressed in log odds probability units (logits) (Bond and Fox, 2007). This is a suitable approach when analyzing ETUQ data because Rasch models can handle the typical scenario that not all ETUQ items are scored as relevant by all participants. That is, the response patterns of all the participants are used by the model to generate individual *person measures*, reflecting the perceived ability in ET use, as well as *item measures*, reflecting the relative challenge of each ET. These measures are displayed in two different hierarchy outputs, one placing the participants on a continuum from lower to higher perceived ability to use ETs, and another one placing the ETs on a continuum from being perceived as less to more challenging to use. A higher person measure indicates higher perceived ability in ET use for a person, while a higher item measure indicates a higher level of perceived challenge for an ET. In this study, special focus was placed on the item measures because our interest was to explore whether the challenge level of certain ETs was more sensitive to cognitive decline. However, we ensured stability and validity of the person measures by securing proper rating scale functioning according to standard procedures described earlier (Nygård *et al*., 2012).

For an ET to be included in the analysis, at least ten participants should have scored it as relevant both at baseline and at year five. This was true for 46 of the 92 items in the ETUQ (50%). In order to detect whether specific ETs presented more, or less, challenge over five-years’ time than expected based upon the Rasch model, we examined differential item functioning (DIF) in relation to time. To adjust for changes in the mean person measure of the sample, actual DIF (Petersson *et al*., 2008) between baseline and year five was calculated for each ET through the use of standardized *z*-comparisons calculated on the individual item measures from both of these time points and their individual standard errors. We used a *z*-score difference of ≥ ±1.96 as the criterion for significant actual DIF, and an item measure of 50.0 logits was used as a cut-off to classify the items as “relatively more easy” or “relatively more challenging” to use at baseline. An item measure of 50.0 logits represents an item where a person with a similar person measure (i.e. 50.0 logits) has an equal chance of scoring a 3 (The ET is sometimes/partly used together with another person) or a 4 (The ET is used with frequent/major difficulties), i.e. the cut-off between using the ET with or without another person. To explore potential patterns in the types of ETs that presented significant actual DIF, the analyzed ETs were also classified into the seven topic areas in which all ETs are organized in the latest ETUQ version (Nygård *et al*., 2016) – home maintenance, information/communication, self-care, maintenance/repair, accessibility, economy/purchasing, and travel.

## Results

At year five, 21 of the 37 included participants contributed with data, which gives a dropout rate of 43%. An independent-sample *t*-test revealed no significant baseline differences regarding age, MMSE, or ETUQ person measure between those who contributed with data at year five and those who had dropped out, and a *χ*^2^ test indicated no significant relation between gender and attrition. The mean perceived ability to use ETs was 54.6 (SD 2.8) logits at baseline and 52.2 (SD 3.1) logits at year five. These person measures were used when adjusting the standardized *z*-score differences examining the actual DIF of the included ETs.

In Table 3, ET item measures of challenge at baseline and year five, standardized *z*-score differences, and item types of the 46 included ETs are presented. In total, seven (15%) of the analyzed ETs had positive *z*-score differences that were outside the ±1.96 interval, i.e. they showed significant actual DIF over time. The positive scores indicate that these ETs were perceived to become more challenging to use over time. For 39 (85%) of the ETs, no significant actual DIF was found, indicating overall significantly stable perceived challenge level over time for these ETs despite overall decreasing ETUQ person measures in the sample. A total of 30 of the 46 analyzed ETs (65%) were classified as “relatively more easy” to use at baseline, while 16 (35%) were classified as “relatively more challenging” to use. At year five, 33 ETs (72%) were classified as “relatively more easy”, while 13 (28%) were classified as “relatively more challenging.” Among the 30 ETs classified as “relatively more easy” to use at baseline, five (17%) – *washing machine*, *TV with remote control*, *hand-held mixer*, *internet interaction*, and *cash machine (ATM)* – showed significant actual DIF, indicating that these became more challenging to use over time. Among the 16 ETs classified as “relatively more challenging” to use at baseline, two (13%) – *stereo/CD player* and *digital camera* – showed significant actual DIF, which reflects that they became even more challenging to use over time.

**Table 3.** Item measures, standardized *z*-score differences examining actual DIF, and ET types for the included ETs, here ordered according to their challenge level at baseline with a cut-off for “relatively more easy” versus “relatively more difficult” to use at 50.0 logits

et (*n*= 46)	item measure in logits at baseline (se)	item measure in logits at year 5 (se)	*z*-score difference between baseline and year 5	et type
Elevator	29.36 (9.88)	20.55 (18.27)	−0.31	Accessibility
Vacuum cleaner	29.60 (9.87)	46.07 (1.70)	1.88	Home maintenance
Answering machine: leave a message when calling	30.11 (9.87)	45.16 (2.33)	1.72	Information/Comm.
Dishwasher	30.57 (9.82)	46.37 (1.79)	1.82	Home maintenance
**Washing machine**	37.45 (6.36)	48.50 (1.38)	**2.07**	**Home maintenance**
Coffee maker/coffee machine	44.89 (2.31)	47.56 (1.66)	1.78	Home maintenance
Ticket-operated queuing system	45.19 (2.16)	44.49 (2.35)	0.54	Accessibility
Stove	46.22 (1.80)	48.14 (1.36)	1.92	Home maintenance
Flushing mechanism in public restroom	46.37 (2.10)	45.82 (2.05)	0.64	Accessibility
Iron	46.43 (1.88)	47.44 (1.49)	1.43	Home maintenance
Electric kettle	46.94 (1.85)	36.67 (8.65)	−0.89	Home maintenance
Automated faucet/dryer in public restroom	47.06 (1.86)	44.11 (2.53)	−0.17	Accessibility
Radio	47.08 (1.80)	46.99 (1.62)	0.96	Information/Comm.
Push-button to get off bus or open bus door	47.11 (1.58)	47.11 (1.84)	0.99	Accessibility
**TV with remote control**	47.28 (1.53)	49.14 (1.25)	**2.16**	**Information/Comm.**
**Hand-held mixer**	47.69 (1.53)	51.14 (1.42)	**2.80**	**Home maintenance**
**Internet interaction**	47.74 (1.69)	51.51 (1.40)	**2.81**	**Information/Comm.**
Calculator	48.04 (1.44)	48.03 (1.79)	1.05	Economy/Purchasing
Microwave oven	48.12 (1.40)	48.31 (1.34)	1.34	Home maintenance
Digital thermometer	48.16 (1.58)	44.01 (2.50)	−0.59	Home maintenance
Egg timer	48.29 (1.48)	43.17 (3.20)	−0.77	Home maintenance
Dryer	48.61 (1.45)	48.99 (1.65)	1.27	Home maintenance
Alarm clock/clock radio	48.81 (1.27)	47.91 (1.54)	0.76	Home maintenance
Toaster	49.19 (1.23)	44.73 (2.79)	−0.67	Home maintenance
Push-button telephone	49.19 (1.28)	42.53 (3.68)	−1.09	Information/Comm.
Cell phone: charger	49.35 (1.21)	46.98 (1.56)	0.02	Information/Comm.
Portable telephone, cordless	49.50 (1.19)	47.64 (1.70)	0.26	Information/Comm.
**Cash machine, ATM**	49.53 (1.21)	51.33 (1.11)	**2.56**	**Economy/Purchasing**
Loyalty card	49.67 (1.24)	49.32 (1.40)	1.10	Economy/Purchasing
Internet information search	49.90 (1.39)	50.33 (1.31)	1.49	Information/Comm.
Cell phone: other	50.01 (1.54)	47.40 (2.14)	−0.08	Information/Comm.
Cell phone: make call	50.14 (1.10)	47.66 (1.45)	−0.04	Information/Comm.
Cell phone: receive call	50.26 (1.10)	46.19 (1.71)	−0.82	Information/Comm.
Credit/debit card with PIN code	50.46 (1.10)	49.86 (1.27)	1.08	Economy/Purchasing
Door code	50.69 (1.06)	48.35 (1.46)	0.04	Accessibility
Smoke detector	50.73 (1.17)	50.51 (1.29)	1.25	Home maintenance
Automated telephone-based functions (e.g. number selector, voice control)	50.97 (1.04)	47.23 (1.69)	−0.67	Information/Comm.
**Stereo/CD player**	51.03 (1.04)	52.29 (1.30)	**2.20**	**Information/Comm.**
Entry phone	51.38 (1.13)	48.14 (1.61)	−0.42	Accessibility
Receiver of digital TV (e.g. converter box)	51.45 (1.30)	52.87 (1.69)	1.80	Information/Comm.
**Camera, digital**	51.52 (1.10)	54.87 (1.64)	**2.92**	**Information/Comm.**
Internet banking	51.52 (1.29)	50.18 (1.73)	0.50	Economy/Purchasing
Remote control: other	52.21 (1.15)	53.21 (1.57)	1.76	Accessibility
Cell phone: text message	52.64 (1.13)	50.17 (1.58)	−0.03	Information/Comm.
DVD	53.01 (0.96)	54.01 (1.50)	1.91	Information/Comm.
Video	55.45 (0.90)	56.35 (1.90)	1.57	Information/Comm.

*Note*: Positive *z*-score difference reflects increasing difficulty of the ET item over time. Negative *z*-score difference reflects decreasing difficulty of the ET item over time. ETs in bold print reach or exceed the ≥ ±1.96 cut-off for significant actual DIF.

As can be seen in Table 3, ET item types within the ETUQ topic areas of self-care, maintenance/repair, and travel are not represented in the analysis due to a small number of scorings.

## Discussion

Our findings show that 15% of the analyzed ETs were significantly more challenging to use at year five compared to at baseline. Based on chance, we would have expected that just over two of the 46 ETs (5%) would demonstrate significant DIF. The findings exceeded this number. However, the majority of the ETs (85%) were perceived to be within a similar challenge level five years after inclusion, despite the fact that the participants’ perceived overall ability to use ETs had decreased and the fact that the diagnostic composition in the sample had changed from 100% MCI to 62% dementia. These findings deserve some reflection.

It is difficult to distinguish common characteristics among the seven ETs that showed significant DIF in this study, in relation to the ETs that did not demonstrate DIF over time. Some of the seven ETs that became significantly more challenging to use could be linked to activities known to reveal early functional signs of cognitive change. For example, perceiving the *cash machine (ATM)* as more challenging to use is in line with earlier findings of impaired ability to manage finances (Arrighi *et al*., 2013), and the increased challenge level in using ETs like *TV with remote control*, *hand-held mixer*, *internet interaction*, *stereo/CD player*, and *digital camera* might be linked to the known early withdrawal from leisure activities in cognitive decline (Arrighi *et al*., 2013; Hedman *et al*., 2016). However, several similar ETs typically used in similar activities were also found among the ETs with stable challenge level, for example, *internet banking*, *receiver of digital TV*, and *internet information search*. Similarly, explanations linked to the complexity of the activities where the ETs were used, or the complexity of the actions involved in the ET use (Malinowsky *et al*., 2011), were not applicable.

The generated hierarchy displaying the ETs on a continuum from less to more challenging to use (Table 3) reaffirms earlier knowledge of domestic ETs as generally being easier to use (Patomella *et al*., 2011; Malinowsky *et al*., 2013) because all but one ET within home maintenance were found among the “relatively more easy” ETs. Somewhat unexpectedly, a larger proportion of ETs classified as “relatively more easy” to use at baseline became significantly more challenging to use at year five (17%) compared to the proportion of ETs with significant DIF that at baseline were classified as “relatively more challenging” (13%). At baseline, the participants likely managed to use the *washing machine*, *TV with remote control*, *hand-held mixer*, *internet interaction*, and *cash machine (ATM)* without the support of another person, albeit with varying degrees of difficulty. By year five, however, several of these ETs had passed over to the “relatively more challenging” side of the 50.0 logit cut-off, thus becoming more likely for the persons to use with another person's support. This finding has at least two clinical implications. First, in order to detect possible functional decline it might be appropriate to ask persons with MCI whether there are ETs that they previously could manage by themselves that now require support from another person. Second, it is relevant to also attend to less challenging ETs within the area of home maintenance and not focus only on high-tech ETs within the areas of information/communication and economy/purchasing because home maintenance involved ETs with significant DIF.

Our finding of a predominantly stable challenge level of ETs over time corresponds in part with a previous study comparing the challenge level of ETs at two time periods three to five years apart in two different samples, including persons with MCI, persons with dementia, and controls (Malinowsky *et al*., 2015). Also, in that study, stable challenge levels were most common with 85% stable ETs. However, contrary to the findings in our study, all but one of the ETs showing a significant change in the level of challenge were perceived as *easier* to use at the later time period in the previous study. This difference might be due to the fact that in the previous study there were different individuals included in the different time periods and the fact that the study also included older adults without cognitive impairments. The direction of the findings in the present study indicates that our underlying assumption might be correct; the challenge levels of certain ETs seem to be more sensitive to cognitive change and might thereby be a useful indicator of functional change in MCI. However, we are reluctant to highlight the specific ETs that showed significant DIF in this study as being, especially sensitive to cognitive decline because no clear patterns regarding their characteristics in comparison to the stable items could be found. We instead propose a comprehensive approach when evaluating the challenge levels of ETs over time in older adults with MCI and dementia, including both easier and more challenging ETs and including domestic ETs as well as ICT.

Given the lack of earlier longitudinal research that explores ET use on the item level in older adults with cognitive decline, this DIF study was undertaken despite the risk of being underpowered. The findings should therefore be interpreted with caution. As a consequence of the large attrition rate of 43%, the sets of persons generating the item measures differ between baseline and year five. However, the dropout analysis shows that the attrition did not differ at baseline from those who contributed with data at year five regarding MMSE, ability to use ETs, gender, or age, which suggests that the generated item calibrations at year five would have been similar without the attrition. Finally, it is important to remember that changes in challenge levels of specific ETs may occur for reasons other than functional change in the participants. For example, updating or replacement of technologies can also contribute to changing challenge levels. Although people with MCI generally are reluctant to replacing ETs with new ones (Nygård, 2008), it is possible that some ETs used by the participants were altered or replaced during the five years. For instance, new features could have made a cell phone easier or more challenging to use. Thus, ET use needs to be interpreted from several perspectives, not only based on the person's characteristics and diagnosis.

In conclusion, the findings of this study suggest that change of perceived challenge of ETs may indicate functional change in persons with cognitive decline, even if alternative explanations cannot be ruled out. Both easier and more challenging ETs typically used at home and in society need to be addressed to capture this functional change because significant changes may occur among ETs of all challenge levels and within all types of ETs in older people with MCI.

## Conflict of interest

None.

## Description of authors’ roles

All authors formulated the research question in consensus. Hedman took part in data collection, performed the statistical analysis, and wrote the paper. Kottorp was responsible for the statistical design of the study, gave advice during the statistical analysis, and provided input to the draft. Almkvist gave advice regarding the statistical analysis and provided input to the draft. Nygård planned the 5-year study, supervised the data collection, and assisted in writing the paper.
